# Acceptability of a computer-tailored and pedometer-based socio-cognitive intervention in a secondary coronary heart disease prevention program: A qualitative study

**DOI:** 10.1177/2055207619899840

**Published:** 2020-01-10

**Authors:** Julie Houle, Maria-Cecilia Gallani, Myriam Pettigrew, Geneviève Laflamme, Luc Mathieu, François Boudreau, Paul Poirier, Sylvie Cossette

**Affiliations:** 1 Nursing Department, Université du Québec à Trois-Rivières, Canada; 2 Faculty of Nursing, Université Laval, Canada; 3 Faculty of Pharmacy, Université Laval, Canada; 4 Faculty of Medicine, Université de Sherbrooke, Canada; 5 Faculty of Nursing, Université de Montréal, Canada

**Keywords:** Computer-tailored, pedometer-based intervention, acceptability study, secondary prevention, socio-cognitive intervention, physical activity

## Abstract

When developing an innovative intervention, its acceptability to patients, health care professionals and managers must be considered to ensure the implementation into practice. This study aims to identify factors influencing the acceptability of a computer-tailored and pedometer-based socio-cognitive intervention for patients with heart disease. Focus group interviews were conducted in two outlying regions of the province of Quebec (Canada). The Theory of Planned Behavior formed the theoretical basis of the interview guide. Two researchers performed verbatim analysis independently until consensus was achieved. The sample included 44 participants divided into six groups (patients *n* = 7 + 8, health care professionals *n* = 8 + 8, managers *n* = 6 + 7). Health care professionals and managers mentioned benefits concerning partners’ opportunity to improve assessment and monitoring. Patients believed the intervention could be useful to improve adherence to physical activity. Additional benefits indicated were self-monitoring behavior and improved health-related outcomes. However, patients expressed concern about the online security, fearing possible data breach. Some clinicians felt the pedometer may not be able to evaluate physical activities other than walking. With regard to behavioral control, a web application and pedometer must be easy to use and compatible with services already in place. Further barriers include level of literacy, cost and the various difficulties associated with wearing a pedometer. Findings suggest that, to improve the acceptability of a computer-tailored and pedometer-based socio-cognitive intervention, users must be assured of a secure website, validated, affordable and easy-to-use pedometers, and an intervention adapted to their level of literacy.

## Introduction

Regular physical activity is a significant protective factor in the primary and secondary prevention of cardiovascular diseases.^1^ In addition, cardiac rehabilitation (CR) is effective for reducing re-hospitalization, morbidity and mortality among patients with cardiac disease.^2,3^ Despite this evidence, long-term adherence to physical activity is a major concern in a secondary prevention program, and few cardiac inpatients enroll in CR programs.^3–5^ Research shows that an outpatient program using a socio-cognitive and pedometer-based intervention effectively increases physical activity level, reduces waist circumference and improves health-related quality of life in the year following an acute coronary disease.^6,7^ Furthermore, computer-tailored interventions are potentially able to improve health behaviors related to chronic conditions, such as physical activity. Computer-tailoring represents “a method of assessing individuals and selecting communication content using data-driven decision rules that produce feedback automatically from a database of content elements” (p.215).^8^

A combined socio-cognitive and pedometer-based intervention delivered through computer-tailoring could help improve physical activity among patients with cardiac disease. Socio-cognitive intervention aims to improve self-efficacy expectation by using different behavior-change techniques.^9^ A pedometer is useful for implementing some behavior-change techniques such as self-monitoring, behavior feedback and goal-setting, while computer-tailoring allows a personalized plan to help a person passing through the different steps leading to adopting and maintaining a behavior (from motivational stage to post-motivational stage while preventing relapse). These have been shown to be the most effective techniques for improving cardiac patients’ physical activity in the post-CR context.^10^ Before developing and evaluating a new and complex intervention, it is worth examining current knowledge on similar interventions and the methods used to evaluate them.^11,12^ The effectiveness of a web-based, computer-tailored, pedometer-based physical activity intervention was evaluated in adults in previous studies.^13,14^ The combination of the pedometer and computer-tailored step advice seems to have the potential to enhance daily step counts, particularly in at-risk persons.^13^ However, to our knowledge, no study has yet been performed combining a computer-tailored and pedometer-based socio-cognitive intervention in CR within the context of secondary prevention programs. These programs aim to increase cardiorespiratory capacity and to reduce cardiovascular risk factors by improving adherence to healthy lifestyles, such as physical activity and diet, as well as medication. Such programs involve the interprofessional collaboration of many health care professionals such as physician, nurse, kinesiologist, physiotherapist and nutritionist.^15^ The health care professionals’ interventions aim at complex set of goals that require the use of participants’ behavioral and cognitive capabilities.^16^

When developing an innovative health care intervention, its acceptability to patients, health care professionals and managers must be evaluated to facilitate future implementation.^17^ This evaluation should provide information on beliefs regarding advantages and disadvantages (attitude), along with facilitating factors and barriers (perceived behavioral control). The present study aims to assess the acceptability of the computer-tailored and pedometer-based (CT-*E*ped) socio-cognitive intervention as a component of CR as well as a secondary prevention program.

## Methods

### Design

A descriptive qualitative study was conducted using focus group methodology to examine the acceptability to participants of a CT-*E*ped socio-cognitive intervention in CR and a secondary prevention program. We used the double-layer design proposed by Krueger and Casey.^18^ Six focus groups (two for each participant category: patients, health care professionals and managers) were conducted to record each actor’s point of view regarding the acceptability of the proposed intervention. Data collection focused on perceived perspectives of the intervention rather than real perspectives of the intervention.

### CT-*E*ped socio-cognitive intervention

The web-based computer-tailoring intervention followed a theory-based development process involving a literature review and a feasibility testing phase, as well as multidisciplinary expertise.^19^ Based on the Theory of Planned Behavior,^20^ Social learning theory^21^ and the Transtheoretical Model,^22^ the CT-*E*ped socio-cognitive intervention includes a number of behavior-change techniques using a web-based computer-tailored intervention combined with a pedometer-based intervention. These interventions have been described previously.^6,23^ The web-based computer-tailored intervention allows action planning and personalized messaging, while the pedometer-based intervention allows objective goal-setting, self-monitoring of behavior and allows feedback on behavior to be given.

The *CT-E*ped socio-cognitive intervention can be applied in an outpatient setting and at home during the year following a hospitalization for a cardiovascular event. In the outpatient setting, the health care professional helps the patient to create a session in the computer-tailored platform and to complete initial data (example: sociodemographic data, health status, behavior stage of change and other relevant data to begin tailoring the intervention). A personalized booklet generated by computer-tailoring is given to the patient. This booklet includes instructions to log on to the computer-tailoring platform at home. Furthermore, the health care professional gives a pedometer to the patient with instructions on how to use it. At home, the patient wears the pedometer daily from morning to bedtime, and downloads the pedometer data in the computer-tailoring platform. The intervention could include three to five motivational sessions based on physical activity-related cognitions and current physical activity level. All sessions may use various techniques associated with the motivational interviewing method and its basic interview skills: (a) asking open questions, (b) affirming, (c) reflecting, (d) summarizing, and (e) informing and advising.^19^ The duration of each session is estimated to be 10–15 min, with a frequency of every 2–4 weeks.

### Study setting and participants

The study was conducted in two general hospitals: one in Trois-Rivières (Mauricie, Quebec, Canada) and the other in Val d’Or (Abitibi-Témiscamingue, Quebec, Canada). Access to the CR center is limited in those regions and they are spread over a vast territory. Three separate groups (for patients, health care professionals and managers) were organized at each site. Between six and eight participants in each group were targeted based on the Krueger and Casey method. A convenience sample was used. The inclusive and exclusive criteria for each group are presented in Table 1. The health care professionals groups included professionals from different health disciplines (e.g., nurses, physicians, kinesiologists) so as to obtain an interdisciplinary perspective. As regards the patient groups, a specific effort was made to include men and women representing different ages and areas (urban vs. rural). The strategies of recruitment used in each group aimed to have samples that are assumed to be logically representative of the target populations. To recruit managers and health care professionals, a research assistant used the lists of employees in order to identify potential participants. Then, an invitation was send to eligible managers and health care professionals. Those who agreed to participate were enrolled up to the targeted sample size. Regarding recruitment of participants in the patient group, a research assistant sent an invitation to those who had given their prior authorization to be contacted regarding a potential research project.

**Table 1.** Selection criteria for each group.

Groups	Inclusion criteria	Exclusion criteria
Patients	Men and women 18 years old or over with ischemic heart disease	Inability to walk at least 10 consecutive minutes Contraindications to physical activity according to the American College of Sport Medicine Coronary bypass surgery in the last 6 months Family or professional relationship to member(s) of research team
Health care professionals	Nurse, physician, kinesiologist, physiotherapist or nutritionist working in cardiac rehabilitation and a secondary prevention program or in an outpatient medical clinic for more than 1 year	Less than 1 year of professional experience in cardiology
Managers	Manager in cardiac rehabilitation and a secondary prevention program or work in a medical clinic for more than 1 year	Less than 1 year of experience as manager

The research assistant phoned or emailed the details to eligible participants and sent them an information document. Participation in focus groups was voluntary. Each patient who took part received $50 CDN as compensation for participation immediately following the group interview. The study was approved by the scientific and research ethics committee of the Centre intégré universitaire de santé et de services sociaux de la Mauricie-et-du-Centre-du-Québec (Project CÉR-2016-004-00) and the research ethics committee of the Université du Québec à Trois-Rivières (Project CER-16-223-07.12).

### Data collection

A group interview was conducted with patients, health care professionals and managers using an interview guide to introduce the topic of discussion. The Theory of Planned Behavior^20^ served as a framework for identifying beliefs likely to influence ongoing acceptability to the intervention. The guide included questions about beliefs regarding advantages and disadvantages (attitude) and facilitating factors and barriers (perceived behavioral control). The principal investigator (JH) began the interview with a brief introduction to explain the rules of the procedure. An example of a CT-*E*ped socio-cognitive intervention was then presented. Next, all participants were invited to give their opinions and discuss the advantages and disadvantages of the intervention (attitude) as well as the facilitating factors and barriers (perceived behavioral control) regarding its implementation. The investigator then concluded the proceedings. The interview lasted 2 hours maximum (Table 2). All sessions were audio-recorded. Each participant was asked to sign a confidentiality agreement and a recording license prior to the session.

**Table 2.** Group interview plan.

	Duration
1. Welcome and introduction of participants	10 minutes
2. Review of objectives and proceedings	5 minutes
3. Explanation of computer-tailored and pedometer-based intervention (CT-*E*ped)	10 minutes
4. Questions and discussion – What are the advantages and disadvantages of the proposed intervention for the secondary prevention of ischemic heart disease? – What persons or groups of persons would approve or disapprove the type of intervention proposed? – Identify possible barriers or facilitators regarding the implementation or use of the proposed intervention. What strategies would counter these barriers or facilitate implementation? – Would you (patients, health care professionals or managers) adhere to the proposed intervention if it was available? – What variables should be considered when analyzing the cost effectiveness of the intervention?	90 minutes
5. Conclusion and acknowledgment	5 minutes

### Analysis

For purposes of data analysis, the audio recording was transcribed verbatim; all transcripts were then organized using Nvivo10 software (Nvivo qualitative data analysis software; QSR International Pty Ltd. Version 10, 2014). Data analysis was conducted based on the Krueger and Casey^18^ content analysis method in which all verbatim transcripts and field notes are read multiple times, then examined for emerging themes. Coding categories were developed, and data were coded and categorized appropriately. The content was then examined to check for missing data, and the material was reviewed. Data were organized based on the study questions. The content of each verbatim transcript was classified into the appropriate themes in order to gather, organize and analyze the information. Classification was consistent with the interview guide. Two research assistants conducted the verbatim analysis, and a double verification was performed with the principal investigator.

## Results

The sample included 44 participants divided into six groups. Sample characteristics are presented in Table 3. Patient groups included men (*n* = 8) and women (*n* = 7). There were more women in the health care professionals groups (13 women vs. three men) and manager groups (11 women vs. two men). The health care professionals groups were composed of nurses and nurses practitioner (*n* = 8), kinesiologist (*n* = 6) and physicians (*n* = 2).

**Table 3.** Samples characteristics (*N*=44).

	Patients	Health care professionals	Managers
	*N*=15	*N*=16	*N*=13
Men (*n*)	8	3	2
Women (*n*)	7	13	11
Age (mean, SD)	72 ±7	–	–
Coronary heart disease	15	–	–
Professional functions			
Nurse (*n*)	–	6	–
Nurse practitioner (*n*)	–	2	–
Kinesiologist (*n*)	–	6	–
Physician (*n*)	–	2	–
Cardiac and/or respiratory services manager (*n*)	–	–	8
Chronic disease program manager (*n*)	–	–	4
Assistant director of nursing (*n*)	–	–	1
Region in Quebec, Canada			
Mauricie (*n*)	8	8	7
Abitibi-témiscamingue (*n*)	7	8	6

The verbatim analysis allowed us to identify patients’, health care professionals’ and managers’ beliefs regarding advantages and disadvantages (attitude) as well as their perception of the facilitating factors and barriers (perceived behavioral control) likely to impact acceptability of the CT-*E*ped socio-cognitive intervention. The main advantages, disadvantages, facilitating factors and barriers for each subgroup of patients, health care professionals and managers are presented in Table 4.

**Table 4.** Themes emerging from verbatim transcripts.

	Number of occurrences (verbatim)			
	Patients	Health care professionals	Managers	Total
*Attitude*				
Advantages				
Promotes motivation and physical activity adherence	8	6	1	15
Enhances evaluation, intervention and follow-up with collaborative approach	1	6	5	12
Self-monitoring	3	1	1	5
Promotes self-management	0	1	3	4
Complements current health care services	0	1	3	4
Provides social support	0	1	2	3
Helps set realistic goals	1	1	0	2
Makes physical activity enjoyable	0	1	0	1
Disadvantages				
Breach of confidentiality	3	1	1	5
Physical activity mainly limited to walking	1	2	0	3
Possible pressure from environment	1	0	1	2
Forgetting to wear the pedometer	1	1	0	2
Inability to measure physical activity in some circumstances	1	0	0	1
*Control perception*				
Facilitating factors				
Easy-to-use Web application and pedometer	4	4	0	8
Compatibility with services already in place	1	3	3	7
Ability to use computers	2	1	2	5
Assistance by health care providers	3	0	1	4
Involvement of health care providers	0	1	2	3
Pedometer free of charge	1	1	0	2
Online transmission of pedometer data	1	0	0	1
Barriers				
Difficulties using technology	3	9	3	15
Cost	1	4	6	11
Internet access	0	0	6	6
Time invested in use of technology (training, management)	0	0	5	5
Wearing pedometer	4	1	0	5
Lack of interest by some professionals	0	1	3	4

### Patient group

The **advantages** most frequently identified by patients are *increased motivation* and *improved self-monitoring of physical activity.* For a better understanding of the results, the terms motivation and self-monitoring will be used to refer to these aforementioned subthemes. Patients mentioned the motivation aspect eight times and emphasized the role of the pedometer as a visual and concrete reminder to help them reach the desired number of steps:

… sometimes I got back in the evening, and well, I didn’t take a whole lot of steps…I’d go out to take a walk. I didn’t walk enough during the day because I had other things to do or … I made myself walk a little at a time just to reach my 10,000 or 12,000 steps a day. (P-AT-03).

Patients also emphasized the importance of a combined pedometer-based and computer-tailored intervention to encourage self-monitoring. They felt this concrete tool enabled them to track their progress and see the benefits of their physical activity practice:

I’ll walk twice a day, but nothing’s counted, so I could stop tomorrow, then I say well I’m doing less exercise, but I have no idea what the problem is … (P-TR-07).

They reported that the most common **disadvantage** was the risk of breach of confidentiality caused by the online transmission of data by health care providers.

Patients identified two key **facilitating factors** associated with computer-tailored intervention: a simple, easy-to-use computer-based platform, which they mentioned three times:

But another thing I’d like is for it to be simple…that we can go online, but simple, really … (P-TR-06)

and a computerized pedometer:

Well in fact, a facilitator, for me, means I wouldn’t have any problem entering my data. (P-AT-07).

Patients stated, again three times, that the intervention would facilitate access to health care providers:

[…], the contribution of a professional, a key practitioner who’s both a motivator and an instructor, and I think that’s important. (P-TR-01).

Regarding the **barriers** perceived, we noted the difficulty of wearing a pedometer at the waist with different types of clothing (e.g., a dress). This concern is voiced by women in the groups:

…because when you’re a girl, like it or not, sooner or later, the pedometer’s not going to work with what you’re wearing  … when you’re wearing a dress, well, obviously it’s not always possible. (P-AT-02).

The difficulty of using computer devices (e.g. computer, iPad, smartphone) is mentioned as a barrier to intervention acceptability. Older patients in the groups are uncomfortable with these technologies and more reluctant to use them:

I use the pedometer, but don’t talk to me about a computer, I’m no good with computers, I don’t want to know anything about them […]. I’m just not…just not a computer person…in fact learning how to use it would be a nightmare because, computers, I can’t even stand thinking about them. (P-AT-04).

### Health care professionals group

Many health care professionals mentioned increasing a patient’s motivation toward physical activity as an **advantage**. They believe that a CT-*E*ped socio-cognitive intervention can serve as a kind of coach to encourage patients’ motivation. The other main advantage indicated is the usefulness of a computerized tool for assessing and monitoring patients’ progress more easily:

Speaking as a physician, this can be an interesting tool for my patients’ follow-up…It could be useful for evaluating further treatment […] So it can be a communication tool, it provides a sense of security for the patient as well … (C-AT-03).

As for the **disadvantages**, health care professionals, particularly kinesiologist, worry that patients will be limited to walking because of the way a pedometer measures physical activity; pedometers are not designed for use during swimming and cycling, for example. Health care professionals also worry about confidentiality and security as regards the online transmission of patient information.

The two **facilitating factors** often mentioned include a web application and easy-to-use pedometer that are compatible with the services already in place, and the ability to use the computer-tailored platform. Health care professionals are sure that a computer-tailored platform would increase the effectiveness of interventions and complement the tools they already use. They also agree that the pedometer offers patients a simple and accessible measurement tool. It is important to keep in mind, however, that the web platform must be as basic and uncomplicated as possible. As for **barriers**, health care professionals mentioned the cost of technologies and the problems they represent for some patients. They believe institutions may not be able to offer pedometers to everyone in the program and question patients’ willingness to purchase their own device:

 … purchasing equipment, in my experience, we wanted to buy blood pressure monitors to lend to patients, but finally […] I don’t think it’s a good idea … to supply them and lend them or find a model that’s less expensive but reliable, since each person can go buy one, obtain one. (C-AT-08)

and:

[…] can people afford to buy this themselves? (C-TR-03)

[…] when we suggested buying the device, the patients said: Oh no, I don’t want to spend money! […] if it’s too expensive … (C-AT-05).

Furthermore, nine health care professionals worry that a key barrier is not only the limited ability of the target population to use web technologies, but also their own ability to guide patients effectively during the intervention:

The more complicated it gets, the less we understand the technologies we have to propose … (C-AT-08)

Me, I don’t consider myself a technology person … (C-AT-08).

### Manager group

Regarding the manager groups, the **advantage** highlighted in the discussion was the improvement of evaluation, patient follow-up and interdisciplinary communications. Furthermore, managers see advantages to promoting self-monitoring and complementing current health care services. As with patients and health care providers, managers worry about protecting confidential information and identify this as a potential **disadvantage** of a web-based intervention.

A **facilitating factor**, according to some managers, is that a compatible platform interface could make health care professionals’ work easier. Finally, managers pointed out more **barriers** than other participants during the focus interviews. The five barriers identified relate to the following subthemes: cost; time required for training and management; difficulty using technology; web access or digital technology; and lack of interest on the part of some providers. Regarding costs and training, managers raised concerns about who will pay for pedometers, since they maintain these devices will not be distributed free of charge in every health care establishment.

[…] we can’t forget that the whole staff will have to be trained […] so that’s going to cost a lot of money. Also, I’m really concerned about the amount of time we’ll have to spend teaching patients. (G-TR-01).

They have doubts about patients’ and health care professionals’ abilities to use this type of intervention:

[…] we have to think about teaching patients to use the technology, obviously, but sometimes we think it’s easy for some of our staff, when in fact it can be a huge problem. (G-TR-05).

Managers voice their concerns about access to the platform. Indeed, workstations in health facilities are secured to ensure confidentiality; complications arise, however, when staff members need to access websites such as the proposed platform:

[…] as for trying to access information, the websites can’t be accessed, they’re secure, so this complicates things too. (G-TR-04).

Finally, an intervention of this kind may not be acceptable to some providers, which means it may be difficult to implement in a different health care setting.

## Discussion

The aim of this study was to assess the acceptability of the CT-*E*ped socio-cognitive intervention as a component of CR as well as a secondary prevention program. Acceptability of health care intervention is a key consideration in the design, evaluation and implementation of an innovative heath care intervention.^17^ Theory of Planned Behavior allow us to explore beliefs about performing a behavior, and is a relevant theory to assess acceptability of healthcare intervention.^17^ The theoretical framework of acceptability proposed that affective attitude, burden, perceived effectiveness, ethicality, intervention coherence, opportunity costs, and self-efficacy are seven components of the acceptability.^17^ Previous studies conducted in adult populations reported good acceptability regarding a new pedometer-based, computer-tailored step advice intervention.^13,14^

Our study provides information on beliefs regarding attitude (advantages and disadvantages) and on perceived behavioral control (facilitating factors and barriers), the latter being conceptually similar to self-efficacy in that both are concerned with perceived ability to perform a behavior.^24^ We found that the attitude toward the CT-*E*ped socio-cognitive intervention in all groups was mainly positive. Indeed, the majority of participants are interested in using this tool either as a treatment or intervention. Based on the focus groups interviews, we identified the main advantages/facilitators and disadvantages/barriers perceived by all parties involved in acceptability of a CT-*E*ped socio-cognitive intervention promoting physical activity adherence in a home-based CR and secondary prevention program.

Patients maintain that this type of intervention increases motivation for physical activity adherence and self-monitoring healthy behaviors. It also facilitates evaluation and follow-up based on objective data. These results are in agreement with literature that reports that interventions including behavior-change techniques derived from control theory (i.e. behavioral goal-setting, action planning, self-monitoring of behavior, feedback on behavior and problem-solving) were associated with greater changes in intention and stage of change than other interventions.^25^ The intervention would complement CR services already in place for patients lacking the time or resources to become fully involved in a center-based CR program. The issue of confidentiality was a crucial concern for all groups and was addressed many times during the discussions. Concern was also expressed about the targeted population’s ability to adapt to the technology. Thus, the need for a secure platform is an important factor, as is the need for educative message adapted to different literacy levels. Health care professionals and managers also highlight possible issues of compatibility with the computer system currently in use in health care centers and hospitals. Finally, managers are concerned with whether or not some patients can afford to purchase their own device.

The literature on CR using mobile technologies has grown over the last decade. Many researchers have examined different devices such as the smartphone or an interactive iPad touch,^26–28^ computers for internet-based programs^29^ or a combination of both.^30^ The pedometer is often employed to measure patients’ physical activity level and a program’s effectiveness.^31^ However, patients, health care professionals and managers have done few qualitative analyses of such programs, and the pedometer has not often been used as part of an intervention. A particular method for planning an effective behavior-change intervention potentially involves a qualitative study based on identifying barriers and facilitators using apps and wearable devices to monitor physical activity behavior.^32,33^ Forman et al. received qualitative feedback from their cohort regarding the mobile smartphone application, *HeartCoach*, used to provide CR.^34^ The intervention required patients to complete a daily to-do list with educational content, medication reminders and physical activity recommendations. Of the 26 patients participating in the program, 83% reported having “a positive overall experience with the *HeartCoach* application,” and 93% said the application “made it easier to adhere to CR activities.” Health care professionals also found it improved adherence and accessibility to the CR program. They mention that the intervention “increased overall quality of CR service,” “communication/connection between visits” and supplied objective data on patients’ behavior. These findings seem to confirm our results regarding the advantages/facilitators identified by our participants. Our proposed intervention differs, however, insofar as we suggest the use of other behavior-change techniques such as goal-setting, behavior self-monitoring, behavior feedback, goal review, action planning, and tailored, personalized messaging. A previous literature review revealed that these techniques were the most effective for increasing physical activity in the post-CR period.^10^

In addition, as far as we know, few studies have been published to date on an intervention that combines a computer-tailored and pedometer-based socio-cognitive intervention. Thorup et al. in Denmark introduced the Teledi@log program, in which a patient was given a Fitbit Zip pedometer, weight scale, sphygmomanometer and tablet with a personal health record interface to communicate with health care providers and provide health information.^35^ Patients wore the pedometer for a 3-month period during which they measured their blood pressure, pulse and weight twice a week. Using self-determination theory, researchers found that the combination of pedometer and tailored program helped satisfy patients’ *autonomy*, *competence* and *relatedness* needs and, at the same time, increased their motivation to engage in walking activities and self-manage their cardiac condition. Another recent study conducted in adults living in Australia support the integration of pedometer into computer-tailored intervention.^36^ This study examined whether a web-based computer-tailored intervention based on theory of planned behavior, self-determination theory and social-cognitive theory, named *TaylorActive*,^37^ combined with pedometer (FitBit Flex) is more effective in increasing physical activity compared with a *TaylorActive* only. The study findings demonstrated that the total physical activity increased more than twice as much in the group using the computer-tailored intervention and pedometer compared with the group using only the computer-tailored intervention. Furthermore, moderate-to-vigorous physical activity increased nearly three times as much at 3 months. Moreover, participants in the pedometer group rated higher acceptability and usability of the web-based computer-tailored intervention than the other group.^36^

### Strengths and limitations

The qualitative descriptive study is an effective preliminary step for discovering the acceptability of a CT-*E*ped socio-cognitive intervention. All parties involved in implementing this intervention (patients, health care professionals and managers) were questioned and felt free to express their opinions. The representative reality of CR far from the main health care centers is relevant. Conducting the study in two regions in Quebec (Canada) enabled us to gather information representing the average of this reality. Although qualitative analyses can sometimes lead to misinterpretation of outcomes owing to de-contextualization of the text, we countered this by having two evaluators perform the analyses separately. It is possible, however, that participants in our sample show a desirability bias. The selection bias related to the recruitment method limits results generalization. This study assessed the acceptability of a CT-*E*ped socio-cognitive intervention in a secondary coronary heart disease prevention program. Future studies, including randomized clinical trials, may offer a promising avenue for verifying its effectiveness for physical activity level and health.

## Conclusion

Our findings suggest that the computer-tailored and pedometer-based socio-cognitive intervention have the potential to be acceptable in a secondary coronary heart disease prevention program. However, to increase the acceptability from patients, health care professionals and managers, the design of the intervention should be addressed. Essential features proposed to improve the acceptability of the CT-*E*ped socio-cognitive intervention include a protected web platform to ensure confidentiality, a component for adapting content to all levels of literacy and an easy-to-use interface. A validated and affordable pedometer, which can be worn on different areas of the body, should also be considered. Thus, all these findings should be used to guide the development of an innovative health care intervention using pedometer and computer-tailoring technology. To increase implementation in the clinical setting, the development of this innovative intervention should be developed in collaboration with knowledge users. Furthermore, a pilot study to evaluate feasibility of this intervention in different contexts is recommended before carrying out a randomized controlled trial to evaluate the efficacy of this intervention.
